# Supplementary material for: Peroxisomal compartmentalization of amino acid biosynthesis reactions imposes an upper limit on compartment size
Source: Nat Commun. 2023 Sep 8;14:5544. doi: 10.1038/s41467-023-41347-x (PMC10491753; doi:10.1038/s41467-023-41347-x)
Supplement: Supplementary file 1 — Supplementary Information [file 41467_2023_41347_MOESM1_ESM.pdf]

Peroxisomal compartmentalization of amino acid biosynthesis reactions imposes an upper limit on compartment size

Supplementary Information

Supplementary figures

Supplementary Figure 1. Gpd2 is required for histidine and lysine biosynthesis.

Supplementary Figure 2. Histidine and lysine biosynthesis enzymes exhibit extensive subcellular compartmentalization.

Supplementary Figure 3. Spontaneous peroxisome loss rescues amino acid biosynthesis in Pex11-deficient cells.

Supplementary Figure 4. Abnormally large peroxisomes over-accumulate metabolic enzymes.

Supplementary Figure 5. Tethering of lysine and histidine biosynthesis enzymes to cytosolic proteins does not disrupt peroxisomal architecture.

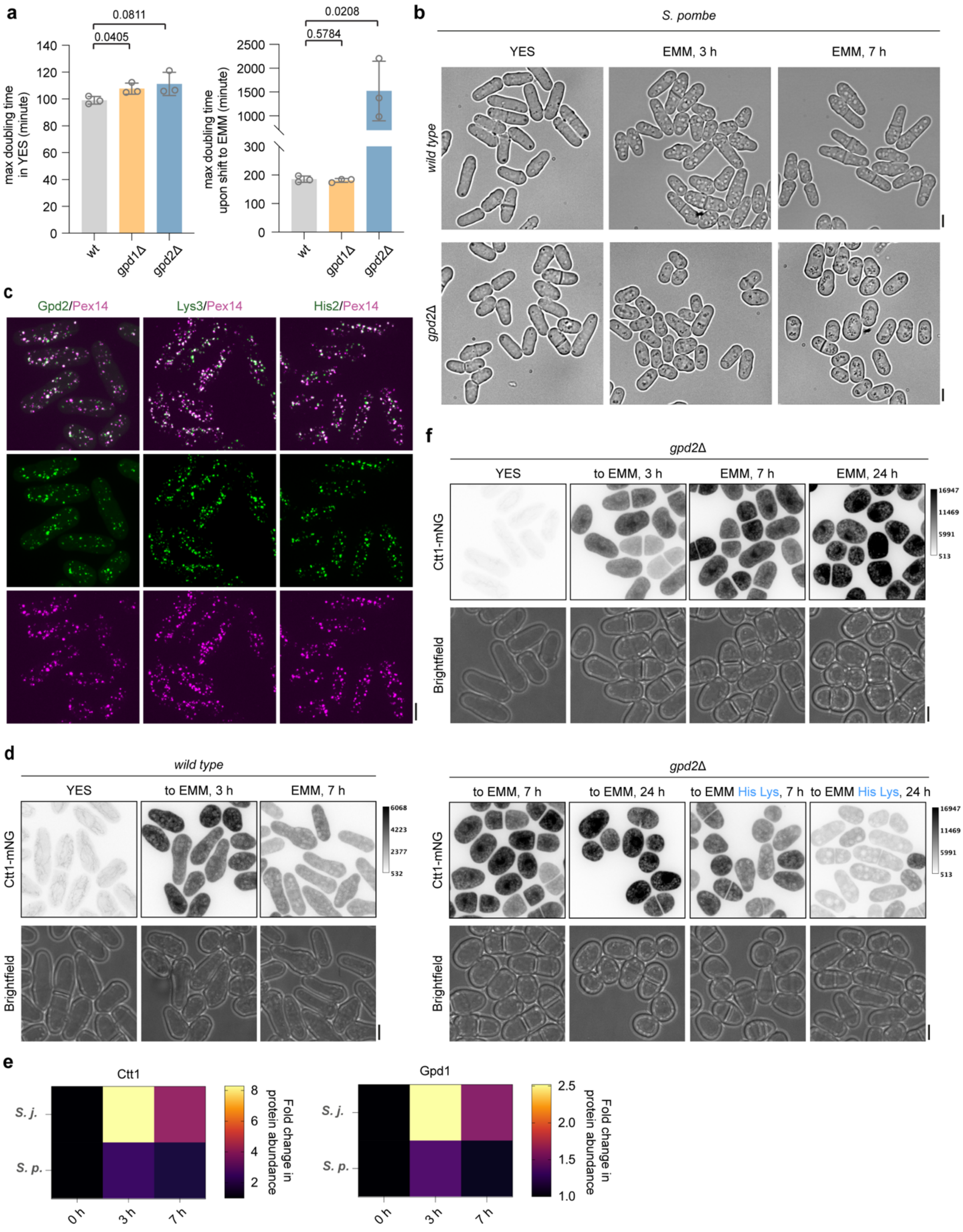

Supplementary Figure 1. Gpd2 is required for histidine and lysine biosynthesis.

a Growth rates of wild type, *gpd1*Δ and *gpd2*Δ *S. pombe* cultures grown in the yeast extract with supplements (YES) (left) and post-shift to the Edinburgh minimal medium (EMM) (right). Bars represent mean values ±SD. *p* values are derived from two-tailed unpaired t-test analysis. **b** Brightfield images of *w*ild type and *gpd2*Δ *S. pombe* cells in YES and following a switch to the minimal EMM medium for 3 h and 7 h. **c** Single channel and colour overlays of maximum Z-projection spinning disk confocal images of *S. japonicus* cells shown in Fig. 1e. **d** Maximum Z-projection spinning disk confocal images of *S. japonicus* expressing Ctt1-mNeonGreen in

indicated conditions. **e** Catalase Ctt1-mNeonGreen (left) and Gpd1-mNeonGreen (right) protein abundance (measured as average intensity of whole cell Z-projection) in *S. japonicus* and *S. pombe* wild type cells sampled at 0, 3 and 7 h time points post-shift to EMM. Heatmap shows ratios between populational means of average cell fluorescence intensity in each dataset. **f** Maximum Z-projection spinning disk confocal images of *gpd2*Δ *S. japonicus* cells expressing Ctt1-mNeonGreen in indicated conditions. Supplemented amino acids L-lysine (Lys) and L-histidine (His) are indicated in blue. Brightfield images are shown underneath the corresponding fluorescence channel images.

(**b-d, f**) Scale bars represent 5 μm. (**a**, **e**) Values are derived from three biological replicates. (**d**, **f**) Greyscale calibration bars are shown, note much higher fluorescence levels of Ctt1-mNeonGreen in **f**. Source data are provided as a Source Data file.

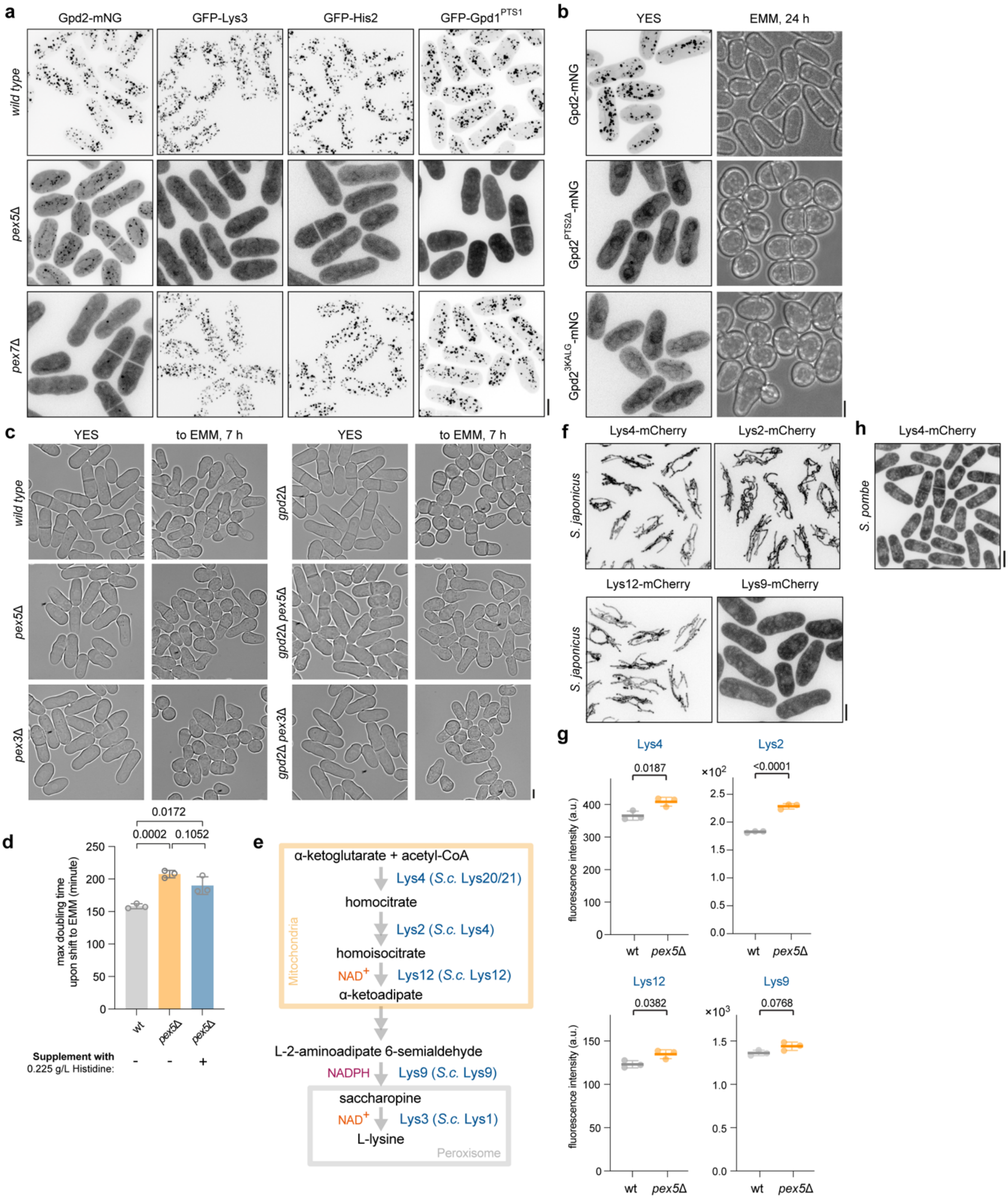

Supplementary Figure 2. Histidine and lysine biosynthesis enzymes exhibit extensive subcellular compartmentalization.

a Maximum Z-projection spinning disk confocal images of wild type, *pex5*Δ and *pex7*Δ *S. japonicus* cells grown in the yeast extract with supplements (YES), expressing Gpd2-mNeonGreen, GFP-Lys3, GFP-His2 or GFP-Gpd1^{PTS1}, respectively. **b** Maximum Z-projection spinning disk confocal images of *S. japonicus* cells expressing mNeonGreen-tagged Gpd2 constructs: wild type Gpd2, a short N-terminal truncation removing PTS2 (Gpd2^{PTS2Δ}), and Gpd2 carrying point mutations in PTS2, which abolish peroxisomal import (Gpd2^{3KALG}) under indicated conditions. **c** Brightfield images of *S. japonicus* cells of indicated genotypes grown in YES and post-shift to the Edinburgh minimal medium (EMM) for 7 h. **d** Growth rates of wild type and *pex5*Δ *S. japonicus* cultures in indicated conditions. **e** Illustration of L-lysine biosynthesis pathway, catalysed by enzymes labelled in dark blue. Assignment of cellular

compartmentalisation of these enzymes is based on fluorescent protein tagging shown in **f** and Fig. 1e. *S. cerevisiae* (*S. c.*) homologs are indicated. Mitochondria are indicated in yellow and peroxisomes in grey. **f** Maximum Z-projection spinning disk confocal images of *S. japonicus* cells expressing mCherry-tagged Lys4, Lys2, Lys12 and Lys9. **g** Protein abundance (measured as average intensity of whole cell Z-projection) of mCherry-tagged enzymes shown in **f** in *S. japonicus* wild type and *pex5*Δ cells sampled at 7 h post-shift to EMM. **h** Maximum Z-projection spinning disk confocal image of *S. pombe* cells expressing mCherry-tagged Lys4.

(**a-c, f, h**) Scale bars represent 5 μm. (**d**, **g**) Values are derived from three biological replicates. *p* values are derived from two-tailed unpaired t-test analysis. Bars represent mean values ±SD. Source data are provided as a Source Data file.

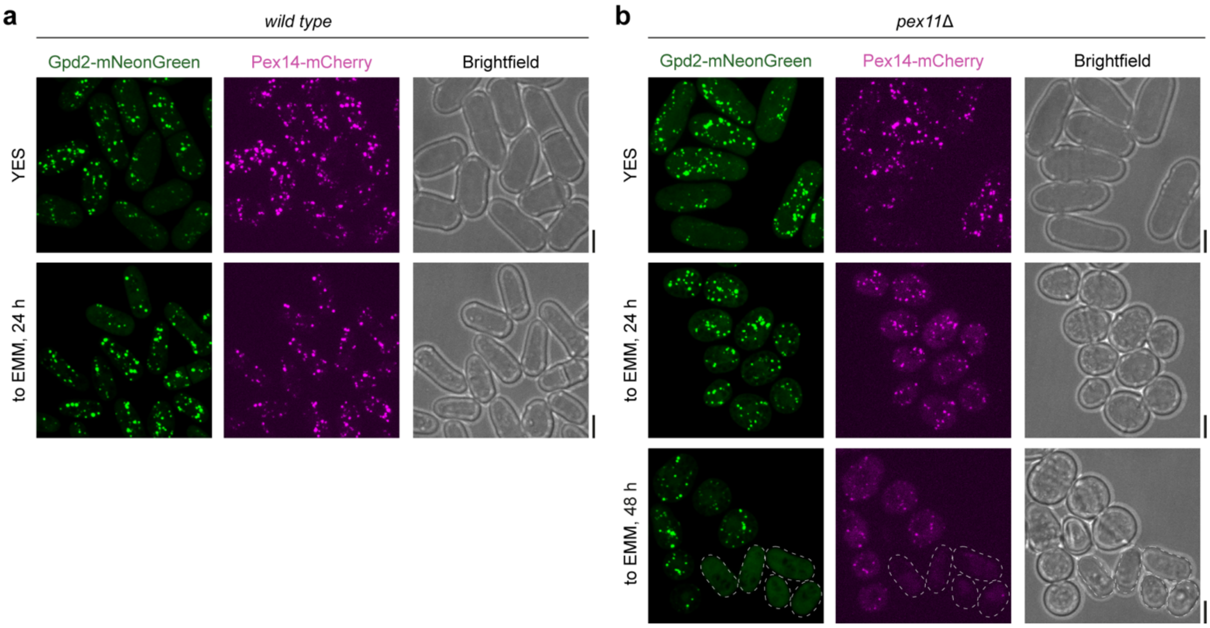

Supplementary Figure 3. Spontaneous peroxisome loss rescues amino acid biosynthesis in Pex11-deficient cells.

a Maximum Z-projection spinning disk confocal images of *S. japonicus* wild type cells co-expressing Gpd2-mNeonGreen (green) and Pex14-mCherry (magenta) grown in the yeast extract with supplements (YES) or post-shift to the Edinburgh minimal medium (EMM) for the indicated time periods. **b** Maximum Z-projection spinning disk confocal images of *S. japonicus pex11Δ* cells co-expressing Gpd2-mNeonGreen (green) and Pex14-mCherry (magenta) grown in YES or post-shift to EMM for the indicated time periods. Cells outlined by a dotted line contain no discernible peroxisomes.

(**a**, **b**) Scale bars represent 5 μm. Brightfield images are shown to the right of the corresponding fluorescence channels.

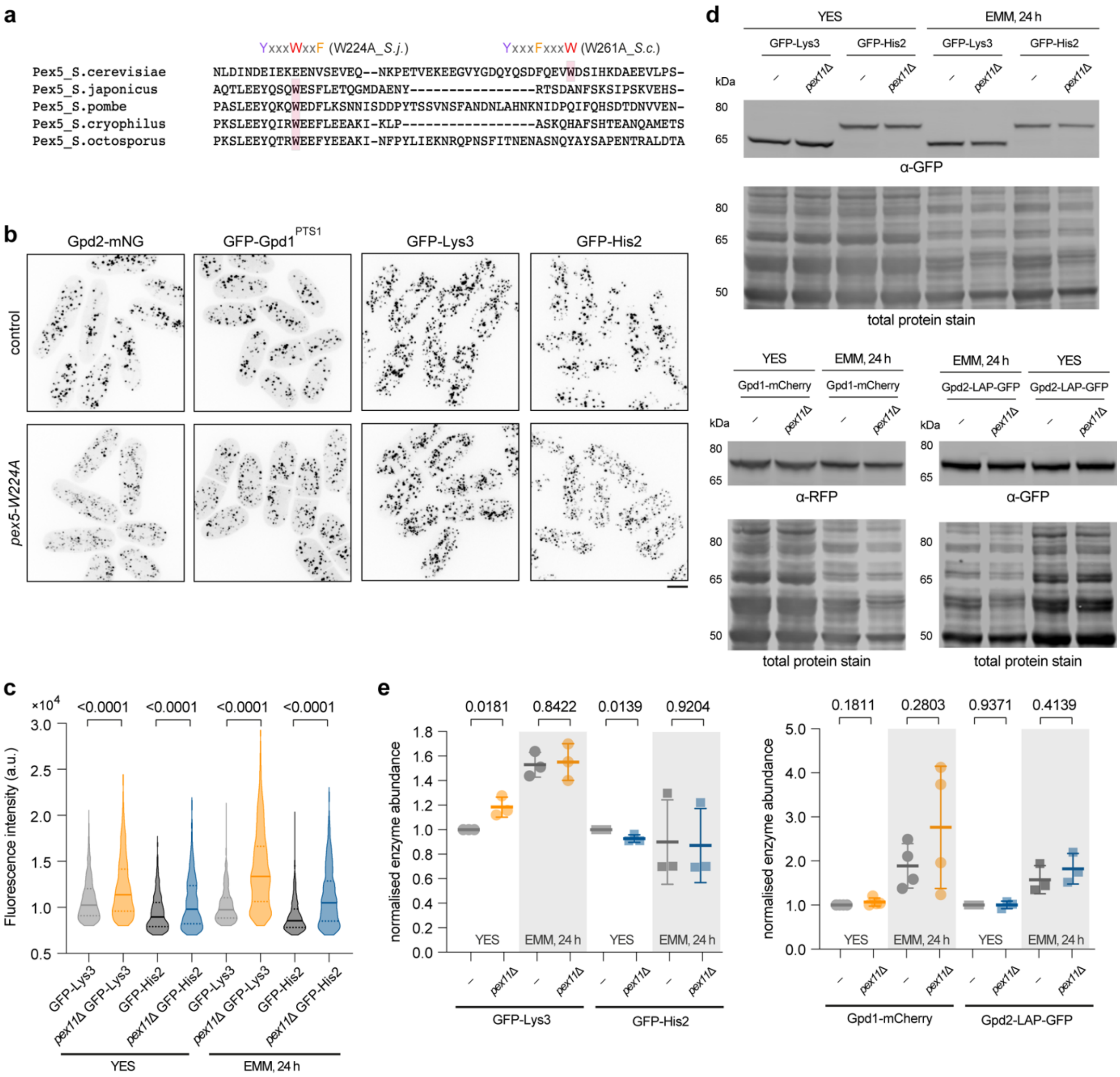

Supplementary Figure 4. Abnormally large peroxisomes over-accumulate metabolic enzymes.

a MUSCLE alignment of peptide sequences spanning the YxxxWxxF motif conserved in Pex5 orthologs from the fission yeast clade, together with the YxxxFxxxW motif in the *S. cerevisiae* Pex5. **b** Maximum Z-projection spinning disk confocal images of wild type and *pex5-W224A S. japonicus* cells expressing Gpd2-mNeonGreen, GFP-Gpd1^{PTS1}, GFP-Lys3 or GFP-His2. **c** Average fluorescence intensities of GFP-Lys3 and GFP-His2, respectively, within peroxisomes of wild type and *pex11Δ* cells grown under indicated conditions. For wild type expressing GFP-Lys3, n=440 and 448 peroxisomes, respectively in the yeast extracts with supplements (YES); n=380 and 389 peroxisomes, respectively in the Edinburgh minimal medium (EMM). For *pex11Δ* expressing GFP-Lys3, n=447 and 434 peroxisomes, respectively in YES; n=346 and 386 peroxisomes, respectively in EMM. For wild type expressing GFP-His2, n=386 and 461 peroxisomes, respectively in YES; n=386 and 376 peroxisomes, respectively in EMM. For *pex11Δ* expressing GFP-His2, n=406 and 443 peroxisomes in respective biological replicate in YES; n=401 and 383 peroxisomes, respectively in EMM. **d** Western blots estimating abundance of GFP-Lys3, GFP-His2, Gpd1-mCherry and Gpd2-LAP-GFP, respectively, detected in whole-cell lysates of wild type and *pex11Δ* cells grown under indicated conditions. **e** Quantification of protein abundance detected by Western blots in **d**.

(**b**) Scale bars represent 5 μm. (**c**) Values are derived from two biological replicates. *p* values are derived from Mann Whitney two-tailed unpaired test. Solid bars represent population medians. Dotted bars represent the upper and lower quartiles. (**e**) Values are derived from at least three biological replicates. *p* values are derived from two-tailed unpaired t-test. Bars represent mean±SD. Source data are provided as a Source Data file.

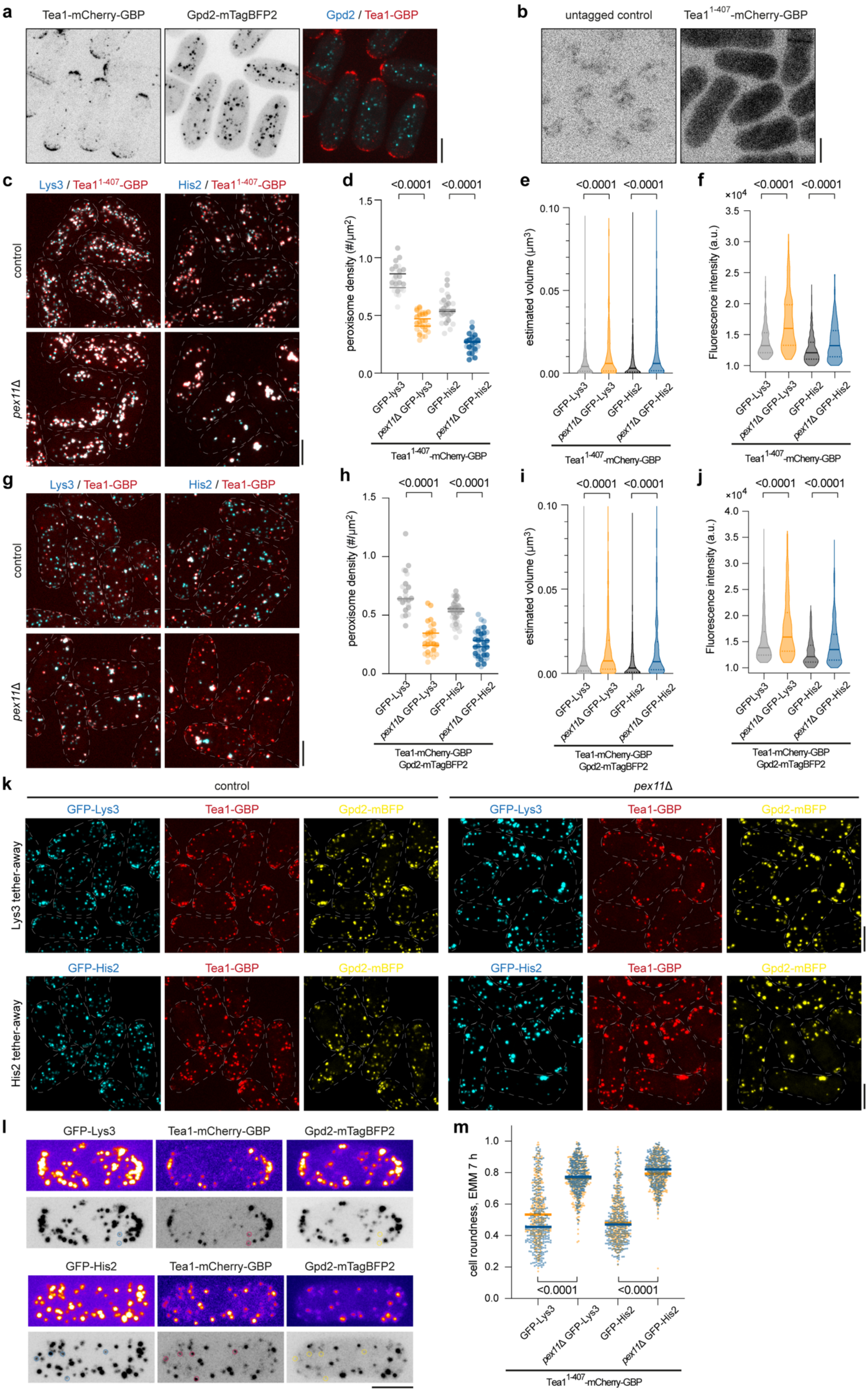
a
Tea1-mCherry-GBP
Gpd2-mTagBFP2
Gpd2 / Tea1-GBP
b
untagged control
Tea1^1-407-mCherry-GBP
c
Lys3 / Tea1^1-407-GBP
His2 / Tea1^1-407-GBP
control
pex11Δ
d
peroxisome density (#/μm²)
<0.0001
GFP-lys3
pex11Δ GFP-lys3
GFP-his2
pex11Δ GFP-his2
Tea1^1-407-mCherry-GBP
e
estimated volume (μm³)
GFP-Lys3
pex11Δ GFP-Lys3
GFP-His2
pex11Δ GFP-His2
f
Fluorescence intensity (a.u.)
×10^4
g
Lys3 / Tea1-GBP
His2 / Tea1-GBP
h
i
j
Tea1-mCherry-GBP
Gpd2-mTagBFP2
k
GFP-Lys3
Tea1-GBP
Gpd2-mBFP
GFP-His2
Lys3 tether-away
His2 tether-away
l
Tea1-mCherry-GBP
m
cell roundness, EMM 7 h

Supplementary Figure 5. Tethering of lysine and histidine biosynthesis enzymes to cytosolic proteins does not disrupt peroxisomal architecture.

a *S. japonicus* cells co-expressing Tea1-mCherry-GBP and Gpd2-mTagBFP2. **b** Wild-type *S. japonicus* and cells expressing Tea1$^{1\text{-}407}$-mCherry-GBP. **c** *S. japonicus* control and *pex11*Δ cells grown in YES, co-expressing Tea1$^{1\text{-}407}$-mCherry-GBP (red) with GFP-Lys3 (cyan) or GFP-His2 (cyan). **d** Peroxisome density shown in **c**. For GFP-Lys3, n=14/14; *pex11*Δ GFP-Lys3, n=14/14. For GFP-His2, n=15/15; *pex11*Δ GFP-His2, n=15/15. **e** Peroxisome size shown in **c**. For GFP-Lys3, n=615/845; *pex11*Δ GFP-Lys3, n=307/471. For GFP-His2, n=774/693; *pex11*Δ GFP-His2, n=310/343. **f** Average fluorescence intensities of GFP-Lys3 or GFP-His2 entities shown in **c**. For GFP-Lys3, n=293/183; *pex11*Δ GFP-Lys3, n=290/201. For GFP-His2, n=239/211; *pex11*Δ GFP-His2, n=298/164. **g** *S. japonicus* control and *pex11*Δ cells grown in YES, co-expressing Tea1-mCherry-GBP (red) with GFP-Lys3 (cyan) or GFP-His2 (cyan). **h** Peroxisome density shown in **g**. For GFP-Lys3, n=15/15; *pex11*Δ GFP-Lys3, n=15/17. For GFP-His2, n=20/20; *pex11*Δ GFP-His2, n=20/21. **i** Peroxisome size shown in **g**. For GFP-Lys3, n=857/831; *pex11*Δ GFP-Lys3, n=418/361. For GFP-His2, n=985/839; *pex11*Δ GFP-His2, n=363/496. **j** Average fluorescence intensities of GFP-Lys3 or GFP-His2 entities shown in **g**. For GFP-Lys3, n=230/200; *pex11*Δ GFP-Lys3, n=190/189. For GFP-His2, n=240/176; *pex11*Δ GFP-His2, n=297/155. **k** Single channel micrographs of cells shown in Fig. 4f. **l** *S. japonicus* cells co-expressing Tea1-mCherry-GBP and Gpd2-mTagBFP2 with either GFP-Lys3 or GFP-His2, shown as individual channels in pseudo-colour and grey. Circles highlight co-localization between GFP-tagged enzymes with Tea1-mCh-GBP lacking Gpd2-mTagBFP2 signal. **m** Cell morphology profiles of indicated genotypes sampled at 7 h post-shift to EMM. For GFP-Lys3, n=321/325; *pex11*Δ GFP-Lys3, n=311/366. For GFP-His2, n=342/314; *pex11*Δ GFP-His2, n=335/347.

(**e**, **f**, **i**, **j**) n denotes peroxisome numbers. (**d**, **h**, **m**) n denotes cell numbers. (**d-f, h-j**, **m**) Values from two biological replicates are separated by the slash symbol. *p* values are derived from Welch's two-tailed unpaired t-test in **d**, **h**, **m** and Mann Whitney two-tailed unpaired test in **e**, **f**, **i**, **j**. Solid bars represent medians. Dotted bars represent the upper and lower quartiles. (**a-c**, **g**, **k**, **l**) Scale bars represent 5 μm. Source data are provided as a Source Data file.
