# Supplementary material for: Peroxisomal compartmentalization of amino acid biosynthesis reactions imposes an upper limit on compartment size
Source: Nat Commun. 2023 Sep 8;14:5544. doi: 10.1038/s41467-023-41347-x (PMC10491753; doi:10.1038/s41467-023-41347-x)
Supplement: Supplementary file 3 — Description of Additional Supplementary Files [file 41467_2023_41347_MOESM3_ESM.pdf]

Description of Additional Supplementary Files

File name: Supplementary Data 1
Description: Fission yeast strains. *S. pombe* and *S. japonicus* strains used in each Figure are listed in Individual spreadsheets.

File name: Supplementary Data 2
Description: Primer sequences. Primers used to generate knockouts, point mutations and fluorescent protein tagging, and genotyping primers are listed in individual spreadsheets.

File name: Supplementary Data 3
Description: Raw metabolomics data. The integrals, as outputted by MANIC/Gavin, are highlighted in pink. The integrals of 4 metabolite mixes (MM, highlighted in green) were used to calculate the amino acid abundances in nmol (highlighted in purple). The m/z values used for quantification of each metabolite and the retention times are shown. Sample legend is provided at the bottom of the spreadsheet.
