# Supplementary material for: Peroxisomal compartmentalization of amino acid biosynthesis reactions imposes an upper limit on compartment size
Source: Nat Commun. 2023 Sep 8;14:5544. doi: 10.1038/s41467-023-41347-x (PMC10491753; doi:10.1038/s41467-023-41347-x)
Supplement: Supplementary file 7 — Reporting Summary [file 41467_2023_41347_MOESM7_ESM.pdf]

nature portfolio

Corresponding author(s): Snezhana Oliferenko
Last updated by author(s): Aug 23, 2023

Reporting Summary

Nature Portfolio wishes to improve the reproducibility of the work that we publish. This form provides structure for consistency and transparency in reporting. For further information on Nature Portfolio policies, see our Editorial Policies and the Editorial Policy Checklist.

Statistics

For all statistical analyses, confirm that the following items are present in the figure legend, table legend, main text, or Methods section.

n/a	Confirmed	
☐	☒	The exact sample size (*n*) for each experimental group/condition, given as a discrete number and unit of measurement
☐	☒	A statement on whether measurements were taken from distinct samples or whether the same sample was measured repeatedly
☐	☒	The statistical test(s) used AND whether they are one- or two-sided<br>*Only common tests should be described solely by name; describe more complex techniques in the Methods section.*
☒	☐	A description of all covariates tested
☒	☐	A description of any assumptions or corrections, such as tests of normality and adjustment for multiple comparisons
☐	☒	A full description of the statistical parameters including central tendency (e.g. means) or other basic estimates (e.g. regression coefficient) AND variation (e.g. standard deviation) or associated estimates of uncertainty (e.g. confidence intervals)
☐	☒	For null hypothesis testing, the test statistic (e.g. *F*, *t*, *r*) with confidence intervals, effect sizes, degrees of freedom and *P* value noted<br>*Give P values as exact values whenever suitable.*
☒	☐	For Bayesian analysis, information on the choice of priors and Markov chain Monte Carlo settings
☒	☐	For hierarchical and complex designs, identification of the appropriate level for tests and full reporting of outcomes
☒	☐	Estimates of effect sizes (e.g. Cohen's *d*, Pearson's *r*), indicating how they were calculated

Our web collection on statistics for biologists contains articles on many of the points above.

Software and code

Policy information about availability of computer code

Data collection	Epifluorescence images were acquired using Zen 2012 (blue edition, Carl Zeiss Microscopy GmbH).<br>Spinning-disk confocal images were acquired using either Andor iQ 3.6.5 (Oxford Instruments) or Andor Fusion 2.3.0.36 (Oxford Instruments).<br>Western blots scans were acquired using Image Studio 5.2 (LI-COR Biosciences).<br>Metabolite data was acquired using MassHunter version B.07.02.1938 (Agilent Technologies).
Data analysis	Fiji v1.53f51 was used for analysis of microscopy images and Western blot scans.<br>YeastSpotter, a web application available at http://yeastspotter.csb.utoronto.ca/, was used for brightfield image segmentation.<br>MassHunter Workstation (Agilent Technologies) and MANIC, an updated version of the software GAVIN [PMID: 21575589], were used for metabolite identification.<br>Prism9 (GraphPad) was used for all statistical analyses.

For manuscripts utilizing custom algorithms or software that are central to the research but not yet described in published literature, software must be made available to editors and reviewers. We strongly encourage code deposition in a community repository (e.g. GitHub). See the Nature Portfolio guidelines for submitting code & software for further information.

Data

Policy information about availability of data

All manuscripts must include a data availability statement. This statement should provide the following information, where applicable:

- Accession codes, unique identifiers, or web links for publicly available datasets
- A description of any restrictions on data availability
- For clinical datasets or third party data, please ensure that the statement adheres to our policy

All data presented in graphs and uncropped scans of all blots generated in this study are included in the Source Data file. Raw metabolomics data is provided in Supplementary Data 3 file. All microscopy and Western blotting data have been deposited in the Figshare database under accession code https://doi.org/10.6084/m9.figshare.c.6798702.v1.

Research involving human participants, their data, or biological material

Policy information about studies with human participants or human data. See also policy information about sex, gender (identity/presentation), and sexual orientation and race, ethnicity and racism.

Reporting on sex and gender	n/a
Reporting on race, ethnicity, or other socially relevant groupings	n/a
Population characteristics	n/a
Recruitment	n/a
Ethics oversight	n/a

Note that full information on the approval of the study protocol must also be provided in the manuscript.

Field-specific reporting

Please select the one below that is the best fit for your research. If you are not sure, read the appropriate sections before making your selection.

☒ Life sciences ☐ Behavioural & social sciences ☐ Ecological, evolutionary & environmental sciences

For a reference copy of the document with all sections, see nature.com/documents/nr-reporting-summary-flat.pdf

Life sciences study design

All studies must disclose on these points even when the disclosure is negative.

Sample size	No sample-size calculations were performed. Sample sizes are based on our experience and community standards. As can be seen from our data, the possible diversity of cellular populations is adequately represented in our samples.
Data exclusions	No data were excluded in the cell biological and physiological experiments. In the measurements of amino acid abundance, linear regression analyses between sample dry weight and amino acid content were performed in Prism 9, for each experimental group. Samples showing anomalous amino acid levels relative to the sample weight were excluded as likely sample preparation artifacts.
Replication	At least two independent biological replicates were performed. All attempts at replication were successful.
Randomization	Samples were allocated based on genotypes of fission yeast strains.
Blinding	The investigators were not blinded to group allocation.

Reporting for specific materials, systems and methods

We require information from authors about some types of materials, experimental systems and methods used in many studies. Here, indicate whether each material, system or method listed is relevant to your study. If you are not sure if a list item applies to your research, read the appropriate section before selecting a response.

Materials & experimental systems

n/a	Involved in the study
☐	☒ Antibodies
☒	☐ Eukaryotic cell lines
☒	☐ Palaeontology and archaeology
☒	☐ Animals and other organisms
☒	☐ Clinical data
☒	☐ Dual use research of concern
☒	☐ Plants

Methods

n/a	Involved in the study
☒	☐ ChIP-seq
☒	☐ Flow cytometry
☒	☐ MRI-based neuroimaging

Antibodies

Antibodies used

Mouse α-GFP (Roche; catalogue no 11814460001, clone IDs: 7.1 and 13.1). Quality check and relevant citations are available at the manufacturer's website (https://www.sigmaaldrich.com/GB/en/product/roche/11814460001).

Mouse α-RFP (Chromotek; catalogue no: 6G6-20, clone ID: 6G6). Validation and relevant citations are available at the manufacturer's website (https://www.ptglab.com/products/RFP-antibody-6G6.htm).

IRDye 800CW goat α-mouse IgG secondary antibody (Li-Cor; catalogue no: 926-32210).

Validation

All primary antibodies were validated for the species. Internal controls include proteins of distinct molecular weight tagged with GFP or mCherry, and non-tagged cellular lysates, shown in the Supplementary Fig. 4d and in uncropped Western blot scans in the Source Data file.
